# Carcass Yields and Meat Composition of Male and Female Italian Slow-Growing Chicken Breeds: *Bianca di Saluzzo* and *Bionda Piemontese*

**DOI:** 10.3390/ani12030406

**Published:** 2022-02-08

**Authors:** Valentina Bongiorno, Achille Schiavone, Manuela Renna, Stefano Sartore, Dominga Soglia, Paola Sacchi, Marta Gariglio, Annelisse Castillo, Cecilia Mugnai, Claudio Forte, Chiara Bianchi, Silvia Mioletti, Laura Gasco, Ilaria Biasato, Alberto Brugiapaglia, Federico Sirri, Marco Zampiga, Francesco Gai, Margherita Marzoni, Silvia Cerolini, Sihem Dabbou

**Affiliations:** 1 Department of Veterinary Sciences, University of Torino, 10095 Grugliasco, TO, Italy; valentina.bongiorno@unito.it (V.B.); achille.schiavone@unito.it (A.S.); manuela.renna@unito.it (M.R.); stefano.sartore@unito.it (S.S.); dominga.soglia@unito.it (D.S.); paola.sacchi@unito.it (P.S.); annelisse.castillogarrido@unito.it (A.C.); cecilia.mugnai@unito.it (C.M.); claudio.forte@unito.it (C.F.); chiara.bianchi@unito.it (C.B.); silvia.mioletti@unito.it (S.M.); 2 Department of Agricultural, Forest and Food Sciences, University of Torino, 10095 Grugliasco, TO, Italy; laura.gasco@unito.it (L.G.); ilaria.biasato@unito.it (I.B.); alberto.brugiapaglia@unito.it (A.B.); 3 Department of Agricultural and Food Sciences, Alma Mater Studiorum, University of Bologna, 40064 Bologna, BO, Italy; federico.sirri@unibo.it (F.S.); marco.zampiga2@unibo.it (M.Z.); 4 Institute of Science of Food Production, National Research Council, 10095 Grugliasco, TO, Italy; francesco.gai@ispa.cnr.it; 5 Department of Veterinary Sciences, University of Pisa, 56124 Pisa, PI, Italy; margherita.marzoni@unipi.it; 6 Department of Veterinary Medicine, University of Milano, 26900 Lodi, LO, Italy; silvia.cerolini@unimi.it; 7 Center Agriculture Food Environment (C3A), University of Trento, 38010 San Michele all’Adige, TN, Italy; sihem.dabbou@unitn.it

**Keywords:** chicken breed, meat quality, sex, carcass yield, fatty acid

## Abstract

**Simple Summary:**

*Bionda Piemontese* and *Bianca di Saluzzo* are two slow growing breeds from northwest Italy, specifically from the Piedmont region. Their low input requirements make them suitable in organic and free-range rearing contexts for both meat and egg production. This research, part of a conservation program for these two breeds, aims to define the meat properties and qualitative attributes of these two breeds, comparing them at different slaughter ages in order to identify the most profitable slaughter period. The results show significant benefits associated with slaughtering at 7 months of age, which outperformed the shorter rearing periods in terms of both better slaughter performances and meat properties.

**Abstract:**

The slaughter performance and meat quality of two native Italian chicken breeds, *Bionda Piemontese* (BP, *n* = 64) and *Bianca di Saluzzo* (BS, *n* = 64), were investigated. Two-way ANOVA, considering breed, sex, and their interaction, was used to compare the properties of birds slaughtered at 5, 6, 7, and 8 months of age. Subsequently, data were analyzed using one-way ANOVA and the Duncan test to evaluate the differences between slaughter ages. The BP breed produced a better carcass yield than BS at 5, 7, and 8 months of age (*p* < 0.05). Breast moisture and crude protein contents were influenced by gender, and were higher in males than in females (*p* < 0.05). By contrast, the crude fat content was higher in females than in males (*p* < 0.05). The saturated fatty acid content of breast meat increased as the birds aged in both breeds (*p* < 0.05). The polyunsaturated fatty acid content of both breast and thigh meat was higher in males than in females (*p* < 0.001 and *p* < 0.05, respectively). In general, slaughtering at 7 months was associated with the best slaughter and meat quality characteristics in both breeds. Moreover, from a nutritional point of view, the characteristics of the meat from male birds were preferable to those of meat from females.

## 1. Introduction

Over the last forty years, the preservation of animal genetic resources has become a matter of great concern, so much so that it was identified by the Food and Agriculture Organization of the United Nations (FAO) as one of its main objectives [1]. Since 1980, technical programs have been developed to gather global census information on all existing livestock breeds and to further activities focused on biodiversity in the livestock sector [1].

The use of just a few commercial poultry hybrids suitable for intensive rearing systems has led to a loss of the 90% of the alleles specific to local poultry breeds, the protection of which has consequently become an argument of great consequence [2,3]. In addition, the conservation of genetic variability is useful for preserving genetic traits that could be successfully used to satisfy future requirements, such as environmental and climate resistant traits [4]. From a commercial point of view, each local poultry breed presents distinct meat qualities, such as, for example, the highly tender and dark meat obtained from the *Padovana* breed. Furthermore, such genotypes are strongly associated with the specific local areas where they have historically been reared, becoming a distinctive social and economic symbol, and reinforcing the concept of poultry biodiversity conservation [5].

According to the Domestic Animal Diversity Information System, a database platform developed and managed by the FAO, 6.74% of the 1482 recognized autochthonous chicken breeds in the world are already extinct. Among the initial 53 Italian chicken breeds, 70% of them are currently extinct and around 20% are now at risk of extinction [6]. At present, Italian indigenous chicken breeds are neither assured nor protected, and for this reason the country’s poultry heritage is becoming increasingly poorer [6].

Different conservation projects have been carried out in Italy over recent years aimed at protecting its native chicken breeds, for example, the “Preserving Biodiversity Project” (BIONET) in the Veneto region [7]; the nation-wide project “Conservation of Biodiversity in Italian Poultry Breeds” (TuBAvi) [8], dedicated to the safeguarding, conservation and improvement of Italian poultry genetic resources; and the project “Germplasm and Agrobiodiversity in Piedmont” (GERMONTE), which employs interventions aimed at characterizing, conserving, and selecting the germplasm of native animal breeds, as well as developing management and breeding plans [9].

A national Italian registry involving 22 native chicken breeds was created and breed standards approved as part of a large cross-sectional conservation project being conducted by the Italian Ministry of Agricultural, Food and Forestry Policies, associated with Ministerial Decree No. 1936 of 1 October 2014 [10].

In recent research carried out by Castillo et al. [6], a questionnaire was administered to Italian breeders aimed at identifying which local breeds were presently being reared across the different Italian regions and to assess the flock numbers of those populations. The two most common native breeds reared were found to be the *Bionda Piemontese* (BP) and the *Livorno* egg-laying hen. On the one hand, some breeds, such as *Collo Nudo Italiano*, *Millefiori Piemontese*, *Pollo Trentino,* and the *Tirolese* chicken, were not listed by breeders at all. Other breeds (*Bianca di Saluzzo* [BS], *Cornuta di Sicilia,* and *Modenese*) were only found to be reared by a small minority of breeders; in fact, only about 20 birds were found for each of these last two breeds. On the other hand, an improving situation was revealed for some breeds that had been studied and that had been the subject of previous conservation projects, such as *Polverara* [6].

From a commercial point of view, local poultry breeds are receiving increasing amounts of consumer interest and represent a smart choice from the biodiversity perspective. Moreover, the use of native chicken breeds has become widely appreciated by consumers in various Western countries, such as France, the UK, the Netherlands, and Germany, in turn, helping to promote genetic variability conservation [11].

Various studies have investigated the meat properties and breed characteristics of Italian chicken breeds with the objective of generating economic interest in these genotypes, obtaining high-quality products for consumers, and promoting genetic biodiversity conservation [5,12,13,14,15].

The BP and the BS breeds are the two local chicken breeds originating from the Piedmont region (Northwest Italy) [16]. The BP breed is characterized by its blond plumage and black tail, while the BS breed is completely white. Nowadays, BP and BS breeds are mainly reared for meat production, although they were formerly considered as dual-purpose breeds (for both egg and meat production) [17]. Usually, BP and BS chickens are slaughtered at approximately 180 days of age since it is the first profitable slaughter period for the farmers. However, in some parts of the Piedmont region, these animals are traditionally reared as seasonal products (consumed mainly during Christmas time), and for this kind of product the rearing period could be longer (up to 8 months). Since 2014, these two poultry breeds have been included in a conservation and genetic improvement program run by the University of Turin and they have also been featured in the Slow Food presidium in 2017, a foundation that sustains quality and endangered productions [17]. The BP and the BS possess behavioral and adaptability traits suitable for organic and free-range rearing systems, as sustained by Ferrante et al. [18] and Soglia et al. [17]. Knowledge about the slaughter performance and meat quality of these two local poultry breeds is lacking, but could prove fundamental for promoting their use and, in turn, their conservation. Moreover, no studies on the slaughter performance and meat quality have been provided for the BS and BP breeds regarding differences among sexes.

The aim of the present study was to characterize slaughter performances and meat quality parameters of BP and BS chickens. By providing data on slaughter performances of these two local chickens breeds (both for male and female), the authors would like to provide important information that can improve the rearing of these genotypes, with an indirect positive effect on the genetic conservation of these Italian breeds. Carcass characteristics, proximate composition, and the fatty acid profile of breast and thigh meat of BP and BS chickens were characterized and compared; moreover, the possible effects of breed, sex, and their interaction were evaluated as a source of variability for the considered traits. These parameters were evaluated at different slaughter ages with the objective of providing information for breeders interested in these two genotypes and to identify a market niche for the products obtained.

## 2. Materials and Methods

The study was carried out at the Avian Conservation Centre for Local Genetic Resources of the University of Turin (Italy) located in Carmagnola (TO), Italy. The Avian Conservation Centre was officially awarded in 2016 by the Italian Ministry of Agriculture and Forestry Policies. The trial was approved by the Bioethical Committee of the University of Turin (Italy) (reference no. 451944, 8 November 2019).

### 2.1. Birds and Husbandry

A total of 320 one-day-old, unsexed chicks from both BS and BP breeds were weighed (average weight 39 g), labelled with a metal wing tag, and reared in indoor pens (2.0 m × 1.0 m, 40 birds/pen, 4 pens/breed) for up to 8 weeks. Birds were kept in a thermoneutral zone and exposed to a 16:8 (light/dark hours) lighting program. At 8 weeks of age, the sex of each bird was identified by direct visual examination. Then, the chicks were separated according to sex, selected on the base of average live weight (LW), and randomly distributed between 16 pens (2.2 m × 3.5 m, 15 birds/pen, 4 pens/sex/breed). From 42 to 240 days of age, all birds had access to outdoor runs (2.2 m × 4.5 m). A total of 240 birds was considered. The pens were equipped with feeders, drinkers, and a suitable shelter to confine chickens at night or during bad weather. From 8 weeks of age onwards, the natural photoperiod was applied (from June, 15:9 light/dark and from November, 9:15 light/dark). All chicks were vaccinated against Marek and Newcastle disease. Chickens had free access to drinking clean and fresh water at all times and were fed ad libitum with a standard commercial starter diet (1–42 days of age) (200 g/kg crude protein, 11.80 MJ metabolizable energy/kg) followed by a growing diet (43–240 days of age) (185 g/kg crude protein, 12.20 MJ metabolizable energy/kg). Clinical signs of illness and mortality were monitored daily throughout the experimental period.

### 2.2. Slaughtering Procedures

Eight males and eight females of each breed and at each slaughter age (5, 6, 7, and 8 months), i.e., 32 birds per age, for an overall total of 128 birds, were selected according to the average final LW in each pen. Each bird was labelled by applying a leg ring and weighed to determine their slaughter weight (SW). The animals were electrically stunned and slaughtered at a commercial abattoir according to standard EU regulations. The plucked and eviscerated carcasses were obtained, and the head, neck, feet, and all abdominal fat removed. Carcasses were stored for 24 h at +4 °C, and then, the chilled carcass (CC) weight was recorded. The CC yield was calculated as a percentage of the SW. Then, the breasts and thighs were excised, and their weights expressed as percentages of the CC weight. A total of 128 breasts and thighs were divided according to the right and left sides, individually vacuum sealed, and refrigerated (4 ± 1 °C). Meat quality parameters (pH 24, color, and drip losses) were assessed on the pectoralis major muscle on the right breast and on the biceps femoris muscle on the right thigh, while the left breast and thigh muscle were freeze-dried and stored at −20 °C until chemical analysis (proximate composition and FA profile).

### 2.3. Meat Quality Parameters

#### 2.3.1. pH

At 24 h post mortem, the pH of the pectoralis major and biceps femoris muscles (under skin, dorsal side) was measured in duplicate using a Crison portable pH meter (Crison Instruments, S.A., Alella, Spain) fitted with a spear-type electrode and an automatic temperature compensation probe.

#### 2.3.2. Color

Breast (pectoralis major, under skin, dorsal side) meat color was measured at 24 h post mortem, using a portable colorimeter Chroma Meter CR-400 Minolta (Minolta Sensing Inc., Osaka, Japan) with an 8 mm diameter measuring area, D65 illuminant, and 2° standard observer. The results were expressed in terms of lightness (L*), redness (a*), and yellowness (b*) in the CIELAB color space (Commission Internationale de l’Éclairage) [19]. Color values were obtained considering the average of three readings per sample.

#### 2.3.3. Drip Losses

At 24 h post mortem, a sample of breast meat was weighed and placed within a container on a supporting mesh and sealed. The samples were blotted for excess surface fluids and re-weighed. Drip losses were determined as percentage of weight lost by the samples during the refrigerated storage period (24 h, at 4 ± 1 °C) [20].

#### 2.3.4. Proximate Composition

Breast and thigh samples were cut, homogenized, and divided into two parts. One portion was used to determine moisture (#950.46) and ash (#920.153) contents according to AOAC International procedures [21]. The remaining part was freeze-dried and analyzed for crude protein (CP) and crude fat (CF) contents, and fatty acid (FA) composition.

In order to estimate the meat CP content, the total N amount was determined, according to the Dumas method [22], using a Macro-N Nitrogen Analyzer (Foss Heraeus Analysensysteme, Hanau, Germany). The CP content was calculated by multiplying the measured nitrogen quantity by the appropriate nitrogen-to-protein conversion factor (6.25). The CF content was determined by Soxhlet extraction with petroleum ether, according to AOAC International method #991.36 [21]. Proximate composition results are expressed as g/100 g of fresh matter (FM).

#### 2.3.5. Hydroxyproline

The collagen content in breast muscle samples was measured indirectly using the method for hydroxyproline (HP) analysis developed by Reddy and Enwemeka [23]. Briefly, 30–50 mg of muscle samples was frozen in liquid nitrogen, and then, lyophilized. The assay employed 2 mL capacity O’-ring screw-capped Nalgene high temperature polypropylene tubes. Aliquots of standard HP or test samples were hydrolyzed in sodium hydroxide (2 N final concentration). Then, the hydrolyzed tissues were mixed with a buffered chloramine-T reagent, and the oxidation was allowed to proceed for 25 min at room temperature. The chromophore was, then, developed by the addition of Ehrlich’s reagent, and the absorbance of the reddish-purple complex was measured at 550 nm using a spectrophotometer. Absorbance values were plotted against the concentration of standard hydroxyproline, and the presence of HP in unknown tissue extracts was determined from the standard curve. HP content is expressed as mg/g dry weight.

#### 2.3.6. Fatty Acid Profile

The FA profile was determined following the method reported by Glass and Cristopherson [24]. Briefly, 250 µg of lipids and 500 µL of a solution of KOH in methanol 2 N were put into a vial containing 5 mL of hexane and 1 g of anhydrous sodium sulphate. The vial was mixed for 30 s and placed in a water bath at 40 °C for 15 min. The sample was cooled on ice after had been stirred. The fatty acid methyl esters (FAME), contained in the upper phase, were collected. Then, they were separated, identified, and quantified using a Shimadzu GC17A gas chromatograph (Shimadzu Corporation, Tokyo, Japan) with a WP-4 Shimadzu integration system equipped with a Varian CPSIL88 capillary column (100 m long, 0.25 mm i.d., 0.20 mm film thickness) (Varian, Walnut Creek, CA, USA) and a flame ionization detector. The gas chromatograph operated in the following conditions: the oven temperature was kept at 170 °C for 15 min, reached 190 °C at a rate of 1 °C/min, then to 220 °C at a rate of 5 °C/min, and kept at this temperature for 17 min. The temperatures of the injector and detector were maintained at 270 °C and 300 °C, respectively. Helium was used as the carrier gas at a constant flow rate of 1.7 mL/min. The individual FAME were identified by comparison with commercial standards (PUFA-2 fatty acid methyl ester standards, Matreya, Pleasant Gap, PA, USA). Quantification was achieved using methyl nonadecanoate 98% (C19:0) (Sigma, Saint Louis, MO, USA) as the internal standard, which was added prior to lipid extraction. The results are expressed as a percentage of each individual FAME per total FAME detected.

### 2.4. Statistical Analysis

The statistical analysis was performed using IBM SPSS Statistics v.27.0 for Windows (IBM SPSS Statistics, Armonk, NY, USA). The effects of breed and gender and their interaction, at each of the four consecutive slaughter ages, on the carcass characteristics, as well as on quality parameters, proximate composition, and FA profile of meat, were analyzed using the following General Linear Model of fixed effects:

Y_ijk_ = μ + Breed_i_ + Sex_j_ + Breed × Sex_ij_ + e_ijk_

where Y_ij_ is the dependent variable, μ is the overall mean, Breed_i_ is the breed effect (i = 1–2), Sex_j_ is the sex effects (j = male and female), Breed × Sex_ij_ is the interaction effect between Breed and Sex, and e_ijk_ is the observational error.

Moreover, a further statistical analyses was performed, adding age at slaughter as a fixed component to the previous model; therefore, when this effect was significant (*p* < 0.05), the Duncan procedure as post hoc test was applied to investigate any differences between the different slaughter ages. The assumption of normality and homogeneity of variance was assessed using Shapiro–Wilk and Levene’s tests, respectively. The results are reported as means plus the standard error of the mean (SEM). Significance is declared for *p* < 0.05.

## 3. Results

With the exception of Table 1, the data reported in the text below represent the average results for the four slaughter ages. Those for the separate slaughter ages are instead reported in the Supplementary Materials.

### 3.1. Slaughter Traits

Table 1 reports the SW and CC weight of BP and BS breeds at each of the four different slaughter ages. In general, a significant effect of gender on the SW and CC weight was observed at each slaughter age (*p* < 0.001). Slaughter age also had a significant effect on SW and CC weight, with the highest values recorded at 7 and 8 months of age (*p* < 0.05), except for BS males, for whom a significant difference in SW was also observed between the two older slaughter ages (being higher at 8 months than at 7 months of age) (*p* < 0.05). Moreover, in BS female birds, the CC weight was unaffected by slaughter age.

The average slaughter traits and breast and thigh yields of the BP and BS chicken breeds are reported in Table 2. As reported above, gender significantly affected the SW and CC weight of the birds (*p* < 0.001). Moreover, the carcass, breast, and thigh yields were affected by gender (*p* < 0.001). Chicken breed did have a significant effect on carcass yield (the lowest value was observed for BS female birds, with an average value of 55.88%, *p* < 0.001).

Specifically, at 8 months of age, as compared with female birds, the males of both breeds showed greater SW (2625.61 ± 33.545 g, average data for the two breeds), CC weights (1522.00 ± 8.485 g), and thigh yields (39.95%) (the average values for females were: 1872.04 ± 18.017 g, 1026.31 ± 65.315 g and 32.40%, respectively, *p* < 0.05) (Table S1).

Moreover, the considered breed significantly affected average carcass yields at 5 (60.25 vs. 58.49% SW for BP and BS, respectively), 7 (59.56 vs. 56.70% SW for BP and BS, respectively), and 8 months of age (57.85 vs. 54.97% SW for BP and BS, respectively, Table S1). Slaughter ages significantly affected carcass yields of BS females, with higher percentages produced at 5 and 6 months of age (*p* < 0.05) (Table S1). However, this parameter did not affect the thigh yield of the two chicken breeds (*p* > 0.05).

Contrary to that observed for the thigh yields, the females of both breeds produced larger breast yields than males (on average 10.62% higher than males, *p* < 0.001) (Table 2). Breast yield was also influenced by the slaughter age of the two chicken breeds, showing the highest values at 5, 7, and 8 months of age for BP female and for BS male and female birds (*p* < 0.05) (Table S1).

### 3.2. Meat Quality Parameters

The average pH (at 24 h from slaughtering), color, and drip loss values for breast and thigh meat are reported in Table 3. Significant differences were observed between the two breeds for the yellowness (b*) of meat, which resulted higher in BP as compared with BS (average values 9.50 vs. 8.72, respectively, *p* < 0.05). Moreover, gender significantly influenced all the evaluated parameters (*p* < 0.05), except for the pH of thigh meat and breast drip losses, which were instead similar between the sexes (*p* > 0.05) (Table 3).

In more detail, gender had little effect on meat pH, with differences only detected at 6 months of age for breast pH and at 5 months of age for thigh pH (*p* < 0.05) (Table S2). The thigh meat pH was significantly affected by the age of female birds (thigh pH in BP females was highest at 7 months of age, whereas in BS females the highest thigh pH values were observed at 6 and 8 months of age, *p* < 0.05), whereas the results in terms of breast meat pH showed no differences at the different slaughter ages, except in BS females (Table S2).

In general, breast lightness (L*), redness (a*), and yellowness (b*) were mainly affected by gender. Higher levels of lightness (L*) and yellowness (b*) were observed in the females of both BP and BS birds, and higher values for redness (a*) were detected in BP and BS males (*p* < 0.001) (Table 3). Specifically, breast lightness (L*) was influenced by gender at 6 and 8 months of age, with higher values in females than males in both the considered breeds (Table S2). Regarding the different slaughter ages, significant differences in breast lightness (L*) were only evident in BS males, with the lowest value observed in birds aged 8 months (*p* < 0.001) (Table S2).

Breast redness (a*) was significantly affected by both gender and slaughter age (*p* < 0.001). Indeed, the females of both BP and BS breeds presented lower redness (a*) levels in breast meat than males at 5, 7, and 8 months of age (*p* < 0.001). Slaughter age had a significant effect on all the birds, with the highest values observed at 8 months of age for this parameter (*p* < 0.05) (Table S2).

Breast yellowness (b*) changed in relation to gender and breed at 6 months of age. Indeed, at 6 months of age, BP showed higher yellowness (b*) values than BS (9.5 vs. 7.99, respectively), and females of both BP and BS showed higher values than males (10.38 vs. 7.12, respectively) (Table S2).

Finally, the average drip loss values were similar between the two breeds and sexes (Table 3). However, when taking the different slaughter ages into consideration, drip losses were significantly influenced by gender at 7 and 8 months of age (*p* < 0.05) (Table S2). Indeed, average drip losses in males were 21.08% greater than in females at 8 months of age (*p* < 0.05) (Table S2). Drip losses were influenced by slaughter age, steadily decreasing as the animals became older (*p* < 0.001) (Table S2).

### 3.3. Breast Meat Chemical Composition and Hydroxyproline Content

The average chemical composition values (moisture, CP, CF, and ash) and hydroxyproline contents of breast meat are summarized in Table 4. Moisture, as well as CF and CP contents were affected by gender (*p* < 0.001). Moisture and CP content were higher in males than in females, whereas the CF content was higher in females than in males (*p* < 0.05) (Table 4).

In more detail, breed did not generally affect the chemical composition of breast meat, except for CF and ash content (at 8 and 6 months of age, respectively, *p* < 0.05) (Table S3). Gender significantly affected moisture (except at 8 months of age) and CF content (*p* < 0.05) (Table S3). At 8 months of age, gender is responsible for a statistical trend for CF content, with the highest values being observed in female birds of the two chicken breeds (*p* = 0.056).

Males showed higher breast moisture and lower CF contents than females (Table S3). Moreover, the CP and ash contents of breast meat were influenced by gender at 5 months of age only (highest CP values in males, lowest ash value in females, *p* < 0.05) (Table S3). Slaughter age mainly affected the moisture and CF contents of breast meat. Higher values of moisture were observed at 5 and 6 months of age for BS and BP males and BP females, respectively. The highest CF value in BP and BS females was observed at 7 months of age. However, in BS males, the highest value was observed at 6, 7, and 8 months of age (*p* < 0.05) (Table S3). Ash content in BS males was also affected by the slaughter age, with the highest values found at 7 and 8 months of age (*p* < 0.05).

Gender affected HP content at 5 months of age (Table S3). Age of slaughter affected HP content in BP females and BS males, with the highest values being observed at 7 and 8 months of age in BS males, and at 5, 6, and 7 months of age in BP females (Table S3).

### 3.4. Thigh Meat Chemical Composition

In general, a significant effect of chicken breed was observed in relation to thigh CP content (being higher in BS than BP), whereas gender significantly affected CP, CF, and ash contents (*p* < 0.05) (Table 5). Indeed, the females showed higher CP and CF contents than males, while lower ash levels were observed in females than in males (*p* < 0.05) (Table 5).

In more detail, gender significantly affected moisture and CF content (*p* < 0.05) (Table S4). Females showed a higher moisture content (+6.80%) than males at 5 months of age. By contrast, moisture content was higher in males than in females at 6, 7, and 8 months of age (*p* < 0.001) (Table S4). Females of both chicken breeds showed higher CF contents at all slaughter ages (*p* < 0.001) (Table S4). The CP content was affected by gender at 6 months of age only, with higher CP levels seen in males than in females.

Finally, regarding the ash content, males had significantly higher levels than females at 5 and 7 months of age (1.08 vs. 0.76 and 1.09 vs. 1.02 g/100 g FM, respectively).

The chemical composition of thigh meat was significantly influenced by slaughter age, since moisture content was significantly higher at 5 months of age as compared with the other slaughter ages in both breeds (*p* < 0.05) (Table S4). On the contrary, the lowest values of thigh CF content were observed at 5 months of age in both chicken breeds (*p* < 0.05) (Table S4).

### 3.5. Fatty Acid Profile of Breast Meat

The overall saturated fatty acid (SFA) composition of breast meat was mainly influenced by gender (*p* < 0.05) (Table 6). In more detail, gender had a significant effect on the total SFA content at 6 and 8 months of age, with male birds showing higher concentrations (*p* < 0.05) (Table S5). Breed affected total SFA content at 7 months of age only, with BP birds showing greater overall SFA levels than BS birds (*p* < 0.05) (Table S5). Slaughter age also affected the total SFA content of breast meat, except for the BS females (*p* < 0.05) (Table S5). SFA levels in BP males increased from 5 to 7 months of age, while no differences were recorded between 7 and 8 months of age. By contrast, the breast SFA content in BS males was higher at 8 months of age as compared with the other slaughter ages (*p* < 0.05) (Table S5). Finally, in BP females, the highest SFA levels in breast meat were observed at 7 months of age (*p* < 0.01) (Table S5).

The averaged data show palmitic acid (C16:0) to be the most abundant SFA in breast meat, the levels of which varied according to breed (being higher in BP than in BS birds) (*p* < 0.05) (Table 6). More specifically, palmitic acid content was influenced by breed at 7 months of age, with the highest value recorded in the BP genotype (*p* < 0.05) (Table S5). At each slaughter age, gender also affected breast palmitic acid content, with females showing higher levels than males (*p* < 0.05) (Table S5). In BP birds of both genders, the palmitic acid content changed in relation to slaughter age, with the lowest values observed at 5 and 6 months (*p* < 0.05) (Table S5).

Stearic acid (C18:0) was the second most abundant FA in breast meat, mainly being affected by gender (higher in males) (Table 6). Specifically, a significant effect of gender on stearic acid content was observed at 6, 7, and 8 months of age. Breed significantly affected breast meat stearic acid content at 7 and 8 months of age, with opposite patterns being observed in BP vs. BS. Indeed, at 7 months of age BP birds showed higher levels of stearic acid than BS (+5.86%, on average), whereas at 8 months of age the opposite was true, with BS birds showing higher levels than BP birds (+9.8%, *p* < 0.05) (Table S5).

The monounsaturated fatty acid (MUFA) content of breast meat was mainly affected by gender, being higher in female birds (*p* < 0.001) (Table 6). Breed had little impact, with BP birds showing a higher MUFA content than BS birds at 8 months of age only (*p* < 0.05) (Table S6). Slaughter age was associated with significant differences. MUFA content in the females of both breeds increased significantly from 6 to 7 months of age (+23.34% and +21.11%, for BP and BS, respectively), whereas it decreased significantly from 7 to 8 months of age (*p* < 0.05) (Table S6). BS male birds showed a lower MUFA content in breast meat at 5 months as compared with all other slaughter ages (*p* < 0.001), whereas no significant evidence was recorded in the BP male group (Table S6).

Oleic acid (C18:1c9) was the most abundant MUFA in breast meat, and was generally higher in female birds as compared with males (*p* < 0.001) (Table 6). Specifically, gender affected breast meat oleic acid levels at 5, 7, and 8 months of age (*p* < 0.001), whereas breed had little effect on this FA, only having an effect at 8 months of age, when it was higher in BP birds as compared with BS birds (*p* < 0.05) (Table S6). Slaughter age was associated with significant differences in oleic acid content. Similar patterns were observed among BP and BS males, which showed the highest oleic acid concentrations at 8 and 7 months of age, respectively (*p* < 0.05) (Table S6). In addition, both BP and BS females exhibited a peak oleic acid content at 7 months of age (*p* < 0.001) (Table S6).

Overall, the Σn6 PUFA content was higher in male than in female birds (*p* < 0.001) (Table 6). In more detail, gender affected breast meat Σn6 PUFA at 5 and 7 months of age, with males showing higher Σn6 PUFA levels than females (*p* < 0.05) (Table S7). The Σn6 PUFA content was significantly higher at 5 months of age as compared with all other slaughter ages (*p* < 0.001) (Table S7).

Linoleic acid (C18:2n6) was the most abundant n6 PUFA in breast meat. It was significantly affected by gender, with the highest levels being observed in males (*p* < 0.05) (Table 6). This FA showed significant differences at 7 months of age, being affected by both breed and gender (*p* < 0.05) (Table S7). Higher linoleic acid levels were observed in male birds of both breeds, although the breast meat of BP birds contained less linoleic acid than in BS birds (*p* < 0.001) (Table S7). A different picture was evident at 6 months of age when BP birds showed higher linoleic acid levels as compared with BS birds (*p* < 0.05) (Table S7). Slaughter age also affected the linoleic acid content of female BP birds, with lower levels recorded at 6 months of age (*p* < 0.001).

Similarly, the average data related to arachidonic acid (C20:4n6) showed differences between the sexes, being higher in males than in females (*p* < 0.05) (Table 6). Moreover, significant differences were also evident among males and females and between the two genders at 5 months of age (*p* < 0.05) (Table S7). Specifically, the males contained more arachidonic acid than the females, and the BP genotype had lower levels as compared with the BS genotype (*p* < 0.05) (Table S7). The breast meat arachidonic acid levels were highest at 5 months of age in all the birds, but had significantly decreased by the time animals were slaughtered at 8 months of age (*p* < 0.001) (Table S7).

The overall results for Σn3 PUFA showed higher levels of these FA in male than in female birds (*p* < 0.05) (Table 6). In particular, the males showed a higher Σn3 PUFA content than females at 5 and 7 months of age (*p* < 0.05) (Table S7). Moreover, the Σn3 PUFA levels were higher in BS birds as compared with BP birds at 7 months of age (*p* < 0.05) (Table S7). Slaughter age significantly affected Σn3 PUFA in both genders of the BP genotype, and BP female birds (*p* < 0.05) (Table S7).

The specific n3 PUFAs considered were: linolenic (C18:3n3), eicosapentaenoic (C20:5n3), docosapentaenoic (C22:5n3), and docosahexaenoic (C22:6n3) acid, each of which was detected in very small amounts in breast meat (Table 6 and Table S7).

In general, the PUFA contents of breast meat were higher in male than in female birds (*p* < 0.001) (Table 6). Considering the data at the different slaughter ages this pattern was confirmed at 5 and 7 months of age (*p* < 0.05) (Table S7). Moreover, the breast meat of BP birds contained less PUFA than the BS group at 7 months of age (*p* < 0.05) (Table S7), and PUFA levels decreased as the birds aged (*p* < 0.05) (Table S7).

### 3.6. Fatty Acid Profile of Thigh Meat

In Table 7, the thigh meat FA profiles of the two breeds of chicken are summarized, as well as the average values of the different slaughter ages considered. The overall total SFA content of thigh meat was affected by both breed and gender. Indeed, a higher SFA amount was detected in BP than BS, and males showed more SFA than females (*p* < 0.001) (Table 7). Breed and the gender both affected the thigh SFA content at 7 and 8 months of age, and the BP thigh meat contained more SFA than the BS genotype (*p* < 0.05) (Table S8).

In general, C16:0 and C18:0 were the most abundant FAs in the thigh meat of both chicken breeds. Breed and gender significantly affected the average palmitic acid (C16:0) content in thigh meat, with BP showing higher levels compared with BS, and higher amount recorded in females than males (*p* < 0.05) (Table 7). Specifically, palmitic acid content was significantly higher in BP birds of 5 and 8 months of age than in BS birds of the same age, and it was always higher in females than in males (*p* < 0.001) (Table S9). Furthermore, a significant incidence effect for the different slaughter ages was only observed in the BS breed, since males showed their highest values at 5, 6, and 7 months of age, whereas in females, the highest values were detected at 6, 7, and 8 months of age (*p* < 0.05) (Table S9).

Gender significantly influenced the average stearic acid (C18:0) levels in thigh meat (being higher in males than in females) (*p* < 0.001) (Table 7). In more detail, gender had a significant incidence on stearic acid content at all slaughter ages, and was always higher in males than in females (*p* < 0.001) (Table S9). Furthermore, only at 5 months of age did breed significantly affect stearic acid content, when levels in the BS genotype were about 11.17% higher than in BP (*p* < 0.05) (Table S9). Slaughter age significantly affected the stearic acid levels in BP males and BS females, with higher levels at 7 and 8 months in BP males, and at 5, 6, and 8 months of age in BS females (*p* < 0.05) (Table S9).

The average MUFA content in thigh meat was higher in females than in males (*p* < 0.001) (Table 7). In more detail, females showed higher MUFA contents than males at all the different slaughter ages (*p* < 0.001) (Table S9). Furthermore, statistical differences were recorded between breeds at 7 months of age, with the BS genotype showing a higher MUFA content than the BP genotype (*p* < 0.05) (Table S9).

Oleic acid (C18:1c9) was the predominant MUFA in thigh meat in all birds, and it was generally affected by the gender of birds, with females showing the highest levels (*p* <0.001) (Table 7). The effects of gender on oleic acid content were detected at all the slaughter ages studied (*p* < 0.05) (Table S9), whereas breed was only found to have a significant effect on thigh oleic acid content at 7 months of age, being higher in the BS genotype as compared with BP birds (*p* < 0.05) (Table S9). Finally, this FA was also influenced by age of slaughter in the BS male group, with significant increases recorded at 6, 7, and 8 months of age (*p* < 0.05) (Table S9).

In general, Σn6 PUFA content was affected by gender, with higher levels recorded in male birds (*p* < 0.001) (Table 7). The gender effect on Σn6 PUFA content could also be observed at different slaughter ages (*p* < 0.05) (Table S10). Moreover, Σn6 PUFA content was influenced by age of slaughter, except in BP male birds (*p* < 0.05) (Table S10). Females of both breeds showed higher values at 5 months of age, while male birds of the BS genotype were instead characterized by an irregular trend (*p* < 0.05) (Table S10).

The most abundant n6 PUFA detected in thigh meat was linoleic acid (C18:2n6), which was significantly affected, however, by gender, being higher in males than in females (*p* < 0.001) (Table 7). In more detail, linoleic acid content varied in relation to gender independently of slaughter age. The age of slaughter influenced thigh linoleic acid content in only the females of either breed, with significantly higher values observed at 5 months of age than the other months considered (*p* < 0.05) (Table S10).

The Σn3 PUFA were the least abundant fatty acids in thigh meat (Table 7). More specially, the only significant difference was observed in the thigh meat from BS females, which was richest in these fatty acids at 5 months of age (*p* < 0.05) (Table S10).

Gender significantly affected the overall PUFA content (*p* < 0.001) (Table 7), being consistently higher in males as compared with females (*p* < 0.05) (Table S10). Statistical differences were observed considering age of slaughter in BP females and in both sexes of the BS genotype. The BP and BS females showed the highest PUFA content at 5 months of age (*p* < 0.05) (Table S10). The highest PUFA values in BS males were recorded at 5 and 8 months of age (*p* < 0.05) (Table S10).

## 4. Discussion

The SW of all birds in the present work increased according to an increase in slaughter age, in agreement with the data reported for BP and BS breeds by Soglia et al. [17]. The SW of BP and BS breeds at 6 months of age was lower than previously reported for the *Milanino* chicken breed slaughtered at that age (which averaged 2843 g and 2318 g for males and females, respectively) [25]. On the contrary, the recorded SW at 5 months of age were slightly higher in BP and BS as compared with those reported for the *Padovana* breed (1882 g for males and 1328 g for females) [26].

Moreover, the CC weight of all birds in the present work increased according to an increase in slaughter age, whereas carcass yields were not influenced by this parameter. Interestingly, the contrary was observed in the BS females, in which the CC weight did not undergo any significant change between 5 and 8 months of age, while the carcass yield was affected by this variable. However, significant differences in terms of carcass yields were observed in relation to breed, even though breed had no significant effect on either SW or CC weight. The better carcass yields observed for BP birds as compared with BS birds (at 5, 7, and 8 months of age) could be linked to more favorable genetic characteristics that should be investigated in future work. Indeed, Zanetti et al. [5] observed significant differences in accordance with genetic traits, in terms of live and carcass weights, between *Padovana*, *Ermellinata,* and *Pepoi* chicken breeds. The recorded carcass yields in the present study were lower than the previously reported results for other Italian chicken breeds, such as the *Romagnola* (62%) and the *Modenese* (63%) breeds [27].

The sexual dimorphism observed in the present study was in line with the findings recorded in BP and BS breeds by Soglia et al. [17]. Similarly, the body weights of the *Géline de Touraine* genotype presented significant differences between males and females, despite similar carcass yields between the two sexes [28]. The higher carcass yields detected in male birds (of both BP and BS) as compared with females in the present work confirmed this gender difference.

Interestingly, the female birds of the BS genotype showed a better carcass yield at 5 and 6 months of age as compared with 7 and 8 months of age, and this result may be related to a later development of the reproductive system and organs that affected the live weight of animals without increasing the carcass weight.

In the current work, the breast yields varied in relation to the slaughter age and gender of the birds. Females displayed higher breast yields than males, whereas males showed higher thigh yields than females. Similar results for breast and thigh yields have been reported by De Marchi et al. [26] in the *Padovana* breed (slaughtered at 150 or 180 days of age) and Tasoniero et al. [29] in the *Padovana* and *Polverara* breeds (slaughtered at 183 days of age). Moreover, Baéza et al. [28] found the same differences between sexes in the *Geline de Touraine*, the *Label rouge* genotype, and in an experimental crossbreed. Indeed, Mignon-Grasteau and Beaumont [30] reported that these differences were related to the precociousness of females over males. Further confirmation came from analysis of the cross-sectional area of muscle fibers, a measure that is directly related to bird age, and which was found to be higher in females than in males when compared at completed body development [28].

Meat color is considered to play a fundamental role in consumer choice, being an important selection criterion for poultry meat and meat products [31]. Consumers generally prefer their poultry meat to have a white or pale tan to pink color [31,32,33]. From a commercial point of view, the meat color of BS and BP breeds could be exploited as a distinctive trait, thus, creating a specific market niche. This objective has already been achieved in some Asian countries. For example, Pongduang et al. [34] demonstrated that red poultry meat with yellow-colored skin had become a feature highly appreciated by consumers in such areas.

The breast meat of both BP and BS females displayed greater lightness than that of male birds, and the value of this parameter also decreased over time in the BS males. On the contrary, Baéza et al. [28] reported no significant differences between sexes in the *Geline de Touraine*, the *Label rouge* genotype, or in an experimental crossbreed.

In the current study, the value of breast meat redness was always greater in male birds as compared with females. In males, breast redness increased as the birds became older, and this could be related to a higher concentration of haeminic pigments that increase with the age of the animals [28,35,36].

In this study, female birds generally showed higher levels of breast yellowness as compared with males. Moreover, differences in breast yellowness were observed in relation to breed. In particular, the breast meat of BP females presented the highest values of yellowness than any other birds at 6 months of age. This could be related both to gender and genetic traits that allow for a higher storage of pigments (such as carotenoids) in fat, which are mainly responsible for the yellow color of poultry meat [28,31,37].

Rizzi et al. [38] reported higher lightness values for the breast meat from *Ermellinata di Rovigo*, *Robusta Lionata,* or *Robusta Maculata* than those obtained in BP or BS breeds at 5–6 months of age. On the contrary, BP and BS breeds showed higher levels of yellowness and redness as compared with the abovementioned breeds [38]. By contrast, higher values in breast lightness, redness, and yellowness were reported for the BP and BS breeds than the *Polverara* and *Padovana* genotypes [29].

Meat color is also related to muscle pH, with a negative trend between the light reflecting properties of meat and pH [39]. Muscle pH also affects the meat’s water holding capacity as well as meat composition [40]. Indeed, low pH values (near to 5.2) are responsible for lower water-holding capacities, being close to the meat isoelectric point that inhibits the capacity of protein to attract water [41]. Moreover, higher pH values have been pointed out as being useful for maintaining favorable meat color and moisture adsorption ability [41].

In the present study, the females of both breeds showed the highest pH values in thigh meat from birds aged 7 months, with the BP genotype associated with higher thigh pH values than the BS genotype.

In this research, the thigh pH values obtained (measured 24 h post mortem) were similar to those found by Devatkal et al. [42] in a slow growing genotype and commercial broilers. Tasoniero et al. [29] reported similar results in terms of breast pH in *Polverara* and *Padovana* breeds (48 h post mortem), while lower values were reported for thigh meat.

With regard to breast drip losses, our results revealed significantly higher values in males at 7 and 8 months of age despite there being no significant differences in terms of breast pH between breeds or genders.

Meat moisture plays a fundamental role in meat quality, being responsible for its perceived juiciness [43]. The moisture content of breast and thigh meat from the BP and BS breeds were similar to those reported by Bogosavljevic-Boskovic et al. [44] for broiler chickens raised in extensive indoor and free-range rearing systems. Interestingly, the moisture content of thigh meat was higher at 5 months of age than at the other slaughter ages considered. This may be related to the increased physical activity and fighting of birds close to sexual maturity that could increase the tonicity of the meat, or could be due to less time spent feeding and consuming water, resulting in the drop in meat moisture content [45]. Dalle Zotte et al. [14] reported similar values of breast meat moisture content in *Polverara* and *Padovana* breeds slaughtered at 6 months of age and BP and BS breeds slaughtered at a similar age.

The CP content of breast meat was only significantly higher in males as compared with in females at 5 months of age. Contrastingly, De Marchi et al. [26] reported similar breast CP content values between male and female *Polverara* and *Padovana* breeds, despite evidence of continuous tissue protein deposition that persisted beyond 5 months of age.

The CF and CP contents of meat have been reported to be affected by chicken strain, developmental growth rate, and bird gender [44]. In the present work, the CF content of thigh meat was higher than that of the breast, and was always higher in females than in males. This corroborates other work showing females to be richer in CF content than males, probably due to their faster tissue growth and rate of fat deposition [28,30,44,46]. Moreover, the important role of oestrogen in promoting lipid synthesis and deposition during the sexual maturity of birds must be considered as underlying the higher CF content of female meat [28].

Hydroxyproline is a secondary amino acid and is found almost exclusively in the connective tissue protein collagen. Consequently, the collagen content of meat can be measured indirectly by measuring hydroxyproline [47]. The HP concentration was significantly higher in BP females and BS males at 7 and 8 months of age. Chuaynukool et al. [48] explained that breed and/or age at slaughter might result in different collagen content levels in chicken meat. The same authors showed that meat from the *Thai* chicken breed at the market age of 16 weeks contained higher total collagen than those of 38-day commercial broilers [48]. Similar findings showing a high collagen content in *Thai* chickens [49] and Korean native chickens [50] as compared with other imported breeds has been reported in the literature. Similarly, Rajkumar et al. [51] showed a higher HP content in *Aseel* chickens as compared with broiler chicken meat at 6 months of age.

Dal Bosco et al. [52] demonstrated differences in meat FA composition in relation to different genotypes. In our study, breed and gender had a significant impact on breast SFA, MUFA, and PUFA contents. The predominant SFAs, MUFAs, and PUFAs found in BS and BP breast meat were the same as those found in five native African chicken genotypes [53].

Dal Bosco et al. [52] reported the BP and BS breeds to have a lower SFA content in breast meat as compared with other slow and medium growing chickens (*Leghorn*, *Ancona*, *Cornish* × *Leghorn*, and *Kabir*). Among the SFAs assessed, breast meat from BP and BS was higher in both palmitic and arachidonic acids as compared with the *Arbor Acres* broiler, the *Chinese* crossbred chicken, and *Hyline* hens [53,54]. Popova et al. [55], however, did not report an overall increase in breast SFA content related to line and age in two lines of slow growing chickens (*La Belle* and *Plymouth Rock*, slaughtered at 9 and 18 weeks of age), although the authors did report an increase in palmitic acid content. By contrast, the BP and BS breeds in the present study did not show any consistent increase in palmitic acid content with age, but a significant increase in total SFA content was recorded in the male birds of both breeds, and the values in males were higher than those in females. This could be related to the shown increase in stearic acid over time, and be due to the fact that the breast stearic acid content was higher in males than in females. These differences between sexes were also reported by Baéza et al. [28].

BP and BS breast meat presented lower levels of PUFA and higher values of MUFA as compared with those of all the above mentioned genotypes. The breast oleic acid content of the BP and BS breeds was similar to that of *Arbor Acres* broilers but lower than that shown in the *Chinese* crossbreed chicken, whereas the linoleic acid content was lower than those reported for all the genotypes studied by Chen et al. [54].

Interestingly, Cerolini et al. [45] reported similar results in male *Milanino* chickens, with an increase in SFA over time, an increase in PUFA content, and a decrease in MUFA content. In the male birds of both breeds in our study, the PUFA content decreased, whereas the MUFA content increased as slaughter age increased.

At all slaughter ages considered, females showed a higher breast MUFA content and a lower breast PUFA content than males. Similar results were observed in the *Géline de Touraine*, *Label rouge,* and an experimental crossbreed studied by Baéza et al. [28]. Klaising [56] reported that birds could synthetize MUFAs, such as oleic and palmitoleic acids. Since female birds are more precocious than males in terms of growth, MUFA synthesis and deposition in the adipose tissue may occur earlier on than in male birds.

The differences in fatty acid content in breast meat in relation to breed, gender, and slaughter age could be useful to identify the best slaughter period in order to obtain the most favorable proportion of fatty acids for human health. This balance is recognized as 1:1.5:1 (SFA/MUFA/PUFA), and is fundamental in order to generate the best LDL/HDL ratio [57]. Based on the results obtained in the present work, the most favorable slaughter age in terms of FA composition in breast meat could be identified at 5 months of age, when the PUFA content was at its highest as compared with the other months studied.

Regarding thigh FA composition, the SFA and MUFA contents were higher than those of breast meat, whereas the PUFA content was lower, due to the lower proportion of linoleic and arachidonic acids.

In the present work, the males were higher in thigh SFA content than females at every slaughter age studied, despite palmitic acid always being higher in females. By contrast, the males presented a higher proportion of stearic acid with respect to females at every age. The BP breed contained more palmitic acid than the BS breed, particularly at 5 months of age, whereas the BS breed contained more stearic acid than the BP breed at this age.

In contrast with the results of the present work, Popova et al. [55] reported that the SFA content in thigh meat was significantly increased in older birds. Chen et al. [54] reported on the FA composition of thigh meat in a Chinese crossbred chicken and *Arbor Acres* broiler, and interestingly, they found lower levels of thigh SFA in these genotypes as compared with those found in the BP and BS breeds. This could be related to the slaughter age of birds that was 45 and 40 days of age for the Chinese crossbred chicken and the *Arbor Acres* broiler, respectively [54]. Therefore, these birds were younger than the BP and BS breeds at the moment of slaughter, and a higher deposition of fat in older birds as compared with younger birds is a well-known fact [58]. Such differences in fat content and FA composition could be explained by the genotype effect that, according to Tang et al. [59], could have a more relevant influence on fat deposition than age of slaughter. Despite differences in total SFA content, the proportion of the palmitic acid content was always greater than that of stearic acid, as also found by Tang et al. [59] and Chen et al. [54].

In general, female birds showed higher thigh MUFA content than males. The BS genotype was higher in MUFA content than the BP genotype at 7 months of age due to increased oleic acid content. The MUFA content in BP and BS genotypes was similar to that of the Chinese crossbreed chicken, but lower than that of the *Arbor Acres* broiler as reported by Chen et al. [54].

Independently of breed, the PUFA content was always higher in males as compared with females. This result lies in accordance with those reported by Cerolini et al. [45] for the *Milanino* chicken breed slaughtered at 7–8 months of age. Thigh PUFA content in female birds was highest at 5 months of age. This could be explained by a lower amount of total CF content at 5 months of age in females. The total CF content increased over time in contrast with the PUFA content that became more diluted. As compared with all the genotypes mentioned by Chen et al. [54], the BP and BS breeds of the present study showed much lower PUFA contents in thigh meat.

## 5. Conclusions

The results obtained in this study suggest that slaughtering at 7 or 8 months of age could present the best carcass parameters in BP birds of both genders and in male BS birds. In addition, 5 and 6 months of age would constitute the best slaughter ages for BS females in terms of carcass yield. Although there were no significant differences in terms of slaughtering weight, the BP genotype appears to be the more productive breed in terms of carcass yield as compared with the BS breed at 5, 7, and 8 months of age. A higher moisture content in thigh meat at 5 months was observed in all birds, and could have a positive influence on the parameter meat juiciness. As compared with the other studies, a lower SFA content and a greater MUFA content were observed in breast meat. Moreover, the BS genotype presented a lower SFA content in thigh meat than the BP genotype. From the health point of view, the breast meat of both BP and BS females was superior to that of their thigh meat due to its lower content of SFA [57].

These results could guide breeders to select the most favorable moment at which to slaughter BS and BP males and females, even though irregular trends were recorded between different slaughter ages in terms of their nutritional profiles and meat properties.

Further research should be conducted to define a stable niche market for BP and BS products and to preserve the genetic variability of these breeds over time. Sensorial analysis could be performed to identify a specific consumer profile and potentially identify any distinctive characteristics of BP and BS meat. Moreover, additional studies should be conducted on meat tenderness and juiciness of these two chicken breeds, since these parameters represent fundamental meat quality indicators for the consumers [60,61].

## Figures and Tables

**Table 1:** Carcass weights of *Bionda Piemontese* and *Bianca di Saluzzo* breeds at different slaughter ages (*n* = 8).

Items	Age	*Bionda Piemontese*		*Bianca di Saluzzo*		SEM	*p*-Value	
		Male	Female	Male	Female		Breed	Gender
SW (g)	5 months	1915.00 ^c^	1505.40 ^c^	1857.46 ^d^	1545.08 ^b^	37.469	0.818	<0.001
	6 months	2187.48 ^b^	1706.58 ^b^	2260.53 ^c^	1695.13 ^ab^	49.017	0.276	<0.001
	7 months	2433.38 ^a^	1808.63 ^ab^	2471.25 ^b^	1928.88 ^a^	65.887	0.345	<0.001
	8 months	2601.89 ^a^	1884.78 ^a^	2649.33 ^a^	1859.30 ^a^	76.416	0.884	<0.001
*p*-Value		<0.001	<0.001	<0.001	0.019			
CC weight (g)	5 months	1148.95 ^c^	910.32 ^b^	1075.70 ^c^	912.24	22.548	0.150	<0.001
	6 months	1325.38 ^b^	1012.50 ^ab^	1342.38 ^b^	978.13	33.044	0.748	<0.001
	7 months	1497.94 ^a^	1042.47 ^a^	1464.27 ^a^	1032.02	44.209	0.585	<0.001
	8 months	1528.00 ^a^	1072.50 ^a^	1516.00 ^a^	980.13	49.622	0.250	<0.001
*p*-Value		<0.001	0.015	<0.001	0.285			

Abbreviations: SW, slaughter weight; CC, chilled carcass; SEM, standard error of the mean. Values with different superscript letters (a–c) within the same column per fixed effect (age of slaughter) differ significantly (*p* < 0.05). The effect of interaction between “Breed” and “Gender” was not significant; therefore, significance is only presented for main effects.

**Table 2:** Average slaughter yields for *Bionda* *Piemontese* and *Bianca di Saluzzo* breeds. Data are the average of the four consecutive slaughterings at 5, 6, 7, and 8 months of age (*n* = 32).

Items	*Bionda Piemontese*		*Bianca di Saluzzo*		SEM	*p*-Value	
	Male	Female	Male	Female		Breed	Gender
SW (g)	2284.43	1726.34	2309.64	1757.09	34.59	0.570	<0.001
CC weight (g)	1375.07	1009.44	1349.58	975.63	21.24	0.277	<0.001
Carcass yield (%SW)	60.28	58.53	58.44	55.88	0.314	<0.001	<0.001
Breast yield (%CC weight)	16.80	19.10	17.29	19.04	0.172	0.455	<0.001
Thigh yield (%CC weight)	39.09	32.57	38.96	32.22	0.697	0.383	<0.001

Abbreviations: SW, slaughter weight; CC, chilled carcass; SEM, standard error of the mean. The effect of interaction between “Breed” and “Gender” was not significant; therefore, significance is only presented for main effects.

**Table 3:** Average meat quality parameters for breast and thigh of *Bionda Piemontese* and *Bianca di Saluzzo* breeds. Data are the average of the four consecutive slaughterings at 5, 6, 7, and 8 months of age (*n* = 32).

Items	*Bionda Piemontese*		*Bianca di Saluzzo*		SEM	*p*-Value	
	Male	Female	Male	Female		Breed	Gender
pH breast	5.91	5.81	5.88	5.84	0.014	0.995	0.010
pH thigh	6.20	6.26	6.26	6.23	0.016	0.533	0.704
Breast L*	51.22	53.90	51.13	54.12	0.271	0.884	<0.001
Breast a*	1.89	−0.05	1.40	−0.61	0.190	0.123	<0.001
Breast b*	8.62	10.39	8.10	9.35	0.201	0.041	<0.001
Breast drip loss (%)	8.12	6.56	7.46	6.57	0.331	0.625	0.067

Abbreviations: L*, lightness; a*, redness; b*, yellowness; SEM, standard error of the mean. The effect of interaction between “Breed” and “Gender” was not significant; therefore, significance is only presented for main effects.

**Table 4:** Average chemical composition (g/100 g FM) and hydroxyproline content (mg/g dry weight) of breast meat of *Bionda Piemontese* and *Bianca di Saluzzo* breeds. Data are the average of the four consecutive slaughtering at 5, 6, 7, and 8 months of age (*n* = 32).

Items	*Bionda Piemontese*		*Bianca di Saluzzo*		SEM	*p*-Value	
	Male	Female	Male	Female		Breed	Gender
Moisture	74.35	73.32	74.16	73.42	0.088	0.789	<0.001
Crude protein	25.00	24.73	25.61	24.84	0.113	0.105	0.021
Crude fat	0.31	0.89	0.22	0.75	0.048	0.177	<0.001
Ash	1.17	1.20	1.17	1.18	0.005	0.308	0.062
Hydroxyproline	2.291	2.548	2.337	2.706	0.198	0.607	0.118

Abbreviations: FM, fresh matter; SEM, standard error of the mean. The effect of interaction between “Breed” and “Gender” was not significant; therefore, significance is only presented for main effects.

**Table 5:** Average chemical composition (g/100 g FM) of thigh meat of *Bionda Piemontese* and *Bianca di Saluzzo* breeds. Data are the average of the four-consecutive slaughtering at 5, 6, 7, and 8 months of age (*n* = 32).

Items	*Bionda Piemontese*		*Bianca di Saluzzo*		SEM	*p*-Value	
	Male	Female	Male	Female		Breed	Gender
Moisture	75.29	74.93	75.41	74.64	0.304	0.893	0.357
Crude protein	27.22	27.53	27.50	28.82	0.181	0.027	0.022
Crude fat	1.71	4.46	1.79	4.34	0.168	0.934	<0.001
Ash	1.11	1.00	1.09	1.00	0.012	0.734	<0.001

Abbreviations: FM, fresh matter; SEM, standard error of the mean. The effect of interaction between “Breed” and “Gender” was not significant; therefore, significance is only presented for main effects.

**Table 6:** Average fatty acid profile for breast meat (g/100 g of total detected fatty acids) of *Bionda Piemotese* and *Bianca di Saluzzo* breeds. Data are the average of the four-consecutive slaughtering at 5, 6, 7, and 8 months of age (*n* = 32).

Items	*Bionda Piemontese*		*Bianca di Saluzzo*		SEM	*p*-Value	
	Male	Female	Male	Female		Breed	Gender
C14:0	0.42	0.64	0.42	0.51	0.052	0.533	0.128
C16:0	28.14	29.41	26.94	27.76	0.294	0.014	0.072
C17:0	0.34	0.31	0.24	0.31	0.020	0.239	0.543
C18:0	14.23	11.61	14.73	12.03	0.172	0.071	<0.001
C24:0	1.07	1.03	1.46	0.90	0.058	0.263	0.009
ΣSFA	44.20	43.01	43.78	41.52	0.349	0.165	0.012
C16:1n7	1.20	2.34	0.90	2.09	0.091	0.062	<0.001
C18:1c9	28.52	35.00	28.18	34.31	0.498	0.535	<0.001
C20:1	0.27	0.20	0.15	0.27	0.017	0.520	0.423
C24:1	0.42	0.50	0.69	0.50	0.038	0.074	0.503
ΣMUFA	30.41	38.05	29.91	37.18	0.563	0.459	<0.001
C18:2n6	13.38	10.91	13.13	11.48	0.313	0.791	0.001
C20:2n6	0.26	0.14	0.29	0.17	0.017	0.402	<0.001
C20:4n6	6.35	4.08	7.05	5.19	0.370	0.213	0.005
Σn6 PUFA	19.99	15.14	20.47	16.88	0.596	0.333	<0.001
C18:3n3	0.08	0.21	0.05	0.17	0.017	0.192	<0.001
C20:5n3	0.45	0.23	0.70	0.33	0.078	0.257	0.059
C22:5n3	0.61	0.37	0.93	0.47	0.043	0.009	<0.001
C22:6n3	0.55	0.64	0.82	0.68	0.050	0.141	0.812
Σn3 PUFA	1.70	1.47	2.50	1.65	0.130	0.054	0.033
ΣPUFA	21.69	16.60	22.97	18.49	0.668	0.217	<0.001
Other FA	3.31	2.21	2.89	2.60	0.157	0.969	0.026

Abbreviations: ΣSFA, total saturated fatty acids; ΣMUFA, total monounsaturated fatty acids; ΣPUFA, total polyunsaturated fatty acids; FA, fatty acids; SEM, standard error of the mean. The effect of interaction between “Breed” and “Gender” was not significant; therefore, significance is only presented for main effects.

**Table 7:** Average fatty acid profile of thigh meat (g/100 g of total detected fatty acids) of *Bionda Piemontese* and *Bianca di Saluzzo* breeds. Data are the average of the four consecutive slaughtering at 5, 6, 7, and 8 months of age (*n* = 32).

Items	*Bionda Piemontese*		*Bianca di Saluzzo*		SEM	*p*-Value	
	Male	Female	Male	Female		Breed	Gender
C14:0	0.89	1.13	0.77	1.00	0.019	<0.001	<0.001
C16:0	32.60	36.24	30.79	34.64	0.254	<0.001	<0.001
C17:0	0.45	0.43	0.46	0.41	0.010	0.810	0.074
C18:0	20.07	12.60	19.98	13.24	0.371	0.497	<0.001
C24:0	0.56	1.07	0.64	0.88	0.041	0.460	<0.001
ΣSFA	54.57	51.47	52.63	50.17	0.274	0.001	<0.001
C16:1n7	2.67	4.50	2.58	4.18	0.103	0.138	<0.001
C18:1c9	34.98	38.19	35.39	39.55	0.287	0.061	<0.001
C20:1	0.40	0.35	0.49	0.39	0.015	0.025	0.010
C24:1	0.18	0.23	0.19	0.15	0.018	0.334	0.975
ΣMUFA	38.24	43.26	38.65	44.28	0.363	0.202	<0.001
C18:2n6	4.38	2.22	4.72	2.17	0.175	0.597	<0.001
C20:2n6	0.02	0.01	0.02	0.01	0.003	0.818	0.028
C20:4n6	0.51	0.21	0.76	0.46	0.064	0.049	0.017
Σn6 PUFA	4.91	2.43	5.50	2.64	0.195	0.204	<0.001
C18:3n3	0.14	0.09	0.13	0.09	0.012	0.679	0.049
C20:5n3	0.03	0.19	0.02	0.06	0.023	0.096	0.023
C22:5n3	0.00	0.03	0.16	0.04	0.026	0.105	0.349
C22:6n3	0.06	0.03	0.23	0.03	0.039	0.263	0.134
Σn3 PUFA	0.23	0.35	0.55	0.21	0.067	0.505	0.408
ΣPUFA	5.14	2.78	6.05	2.85	0.210	0.152	<0.001
Other FA	2.04	2.48	2.66	2.71	0.129	0.105	0.343

Abbreviations: ΣSFA, total saturated fatty acids; ΣMUFA, total monounsaturated fatty acids; ΣPUFA, total polyunsaturated fatty acids; FA, fatty acids; SEM, standard error of the mean. The effect of interaction between “Breed” and “Gender” was not significant; therefore, significance is only presented for main effects.

## Data Availability

The data presented in this study are available on request from the corresponding author.

## Supplementary Materials

The following supporting information can be downloaded at: https://www.mdpi.com/article/10.3390/ani12030406/s1, Table S1: Slaughter yields of *Bionda Piemontese* and *Bianca di Saluzzo* breeds at different slaughter ages, Table S2: Meat quality parameters of breast and thigh muscles at different slaughter ages, Table S3: Chemical composition (g/100 g FM) and hydroxyproline content (mg/g dry weight) of breast meat at different slaughter ages, Table S4: Chemical composition (g/100 g FM) of thigh meat at different slaughter ages, Table S5: Saturated fatty acid profile of breast meat at different slaughter ages (g/100 g of total fatty acids), Table S6: Monounsaturated fatty acid profile of breast meat at different slaughter ages (g/100 g of total fatty acids), Table S7: Polyunsaturated fatty acid profile of breast meat at different slaughter ages (g/100 g of total fatty acids), Table S8: Saturated fatty acid profile of thigh meat at different slaughter ages (g/100 g of total fatty acids), Table S9: Monounsaturated fatty acid profile of thigh meat at different slaughter ages (g/100 g of total fatty acids), Table S10: Polyunsaturated fatty acid profile of thigh meat at different slaughter ages (g/100 g of total fatty acids).
