# Supplementary material for: Carcass Yields and Meat Composition of Male and Female Italian Slow-Growing Chicken Breeds: Bianca di Saluzzo and Bionda Piemontese
Source: Animals (Basel). 2022 Feb 8;12(3):406. doi: 10.3390/ani12030406 (PMC8833521; doi:10.3390/ani12030406)
Supplement: Supplementary file 1 [file animals-12-00406-s001.zip › animals-1557162-supplementary.pdf]

animals

MDPI

Article

Carcass Yields and Meat Composition of Male and Female Italian Slow-Growing Chicken Breeds: *Bianca di Saluzzo* and *Bionda Piemontese*

Valentina Bongiorno[1], Achille Schiavone[1], Manuela Renna[1], Stefano Sartore[1], Dominga Soglia[1], Paola Sacchi[1], Marta Gariglio[1]*, Annelisse Castillo [1], Cecilia Mugnai[1], Claudio Forte[1], Chiara Bianchi[1], Silvia Mioletti[1], Laura Gasco[2], Ilaria Biasato[2], Alberto Brugiapaglia[2], Federico Sirri[3], Marco Zampiga[3], Francesco Gai [4], Margherita Marzoni[5], Silvia and erolini[6], Sihem Dabbou[7]

[1]Department of Veterinary Sciences, University of Torino, 10095 Grugliasco, TO, Italy; valentina.bongiorno@unito.it (V.B.); achille.schiavone@unito.it (A.S.); manuela.renna@unito.it (M.R.); stefano.sartore@unito.it (S.S.); dominga.soglia@unito.it (D.S.); paola.sacchi@unito.it (P.S.); annelisse.castillogarrido@unito.it (A.C.); cecilia.mugnai@unito.it (C.M.); claudio.forte@unito.it (C.F.); chiara.bianchi@unito.it (C.B.); silvia.mioletti@unito.it (S.M.)

[2] Department of Agricultural, Forest and Food Sciences, University of Torino, 10095 Grugliasco, TO, Italy; laura.gasco@unito.it (L.G); ilaria.biasato@unito.it (I.B); alberto.brugiapaglia@unito.it (A.B)

[3]Department of Agricultural and Food Sciences, Alma Mater Studiorum – University of Bologna, Ozzano dell'Emilia, 40064 Bologna, BO, Italy; federico.sirri@unibo.it(F.S); marco.zampiga2@unibo.it (M.Z)

[4]Institute of Science of Food Production, National Research Council, 10095 Grugliasco, TO, Italy; francesco.gai@ispa.cnr.it (F.G)

[5]Department of Veterinary Sciences, University of Pisa, 56124 Pisa, PI, Italy; margherita.marzoni@unipi.it (M.M.)

[6]Department of Veterinary Medicine, University of Milano, 26900 Lodi, LO, Italy; silvia.cerolini@unimi.it (S.C.)

[7]Center Agriculture Food Environment (C3A), University of Trento, 38010 San Michele all'Adige, TN Italy; sihem.dabbou@unitn.it (S.D)

*Correspondence: marta.gariglio@unito.it

Supplementary materials

Table S1. Slaughter yields of *Bionda Piemontese* and *Bianca di Saluzzo* breeds at different slaughter ages (n = 8).

Items	Age at slaughter	*Bionda Piemontese*		*Bianca di Saluzzo*		SEM	*p*-value	
		Male	Female	Male	Female		Breed	Gender
Carcass yield (% SW)	5 months	60.03	60.47	57.96	59.03[a]	0.431	0.028	0.326
	6 months	60.58	59.33	59.38	57.58[ab]	0.490	0.131	0.119
	7 months	61.58	57.54	59.20	54.21[bc]	0.761	0.029	0.001
	8 months	58.92	56.78	57.22	52.72[c]	0.678	0.017	0.007
***p*-value**		0.080	0.083	0.122	0.020			
Breast yield (%CC)	5 months	16.16[b]	19.09[a]	17.76[a]	19.02[b]	0.285	0.064	<0.001
	6 months	15.45[b]	17.39[c]	15.65[b]	16.87[c]	0.228	0.658	<0.001
	7 months	17.50[a]	18.92[bc]	17.72[a]	19.63[ab]	0.290	0.371	0.003
	8 months	18.09[a]	20.99[a]	17.91[a]	20.67[a]	0.324	0.556	<0.001
***p*-value**		<0.001	0.002	0.002	<0.001			
Thigh yield (%CC)	5 months	38.89	33.23	38.56	32.67	0.626	0.544	<0.001
	6 months	39.03	32.46	38.41	31.59	0.654	0.118	<0.001
	7 months	38.51	32.17	38.93	32.25	0.621	0.573	<0.001
	8 months	39.95	32.41	39.96	32.39	0.714	0.989	<0.001
***p*-value**		0.385	0.513	0.230	0.480			

Abbreviations: SW: slaughter weight; CC: chilled carcass weight; SEM: standard error of the mean; values with different superscript letters (a, b, c) within the same column per fixed effect (age of slaughter) differ significantly ($p < 0.05$). The effect of interaction between "Breed" and "Gender" was not significant; therefore, significance is only presented for main effects.

Table S2. Meat quality parameters of breast and thigh muscles at different slaughter ages (n = 8).

**Items**	**Age at slaughter**	***Bionda Piemontese***		***Bianca di Saluzzo***		**SEM**	***p*-value**	
		**Male**	**Female**	**Male**	**Female**		**Breed**	**Gender**
pH breast	5 months	6.21	6.10^{b}	6.34	6.15^{c}	0.035	0.189	0.034
	6 months	6.18	6.25^{b}	6.21	6.24^{ab}	0.019	0.823	0.187
	7 months	6.22	6.45^{a}	6.22	6.21^{c}	0.031	0.034	0.052
	8 months	6.21	6.24^{b}	6.31	6.35^{a}	0.037	0.185	0.650
***p*-value**		0.974	0.002	0.512	0.015			
pH thigh	5 months	6.21	6.10^{b}	6.34	6.15^{c}	0.035	0.189	0.034
	6 months	6.18	6.25^{b}	6.21	6.24^{ab}	0.019	0.823	0.187
	7 months	6.22	6.45^{a}	6.22	6.21^{c}	0.031	0.034	0.052
	8 months	6.21	6.24^{b}	6.31	6.35^{a}	0.037	0.185	0.650
***p*-value**		0.974	0.002	0.512	0.015			
			Breast meat					
Lightness (L*)	5 months	51.69	53.98	52.54^{a}	53.66	0.514	0.800	0.108
	6 months	50.10	53.15	50.98^{a}	54.45	0.503	0.200	0.001
	7 months	52.96	55.05	53.47^{a}	54.03	0.403	0.750	0.106
	8 months	50.12	53.42	47.55^{b}	54.36	0.644	0.366	<0.001
***p*-value**		0.102	0.447	<0.001	0.931			
Redness (a*)	5 months	-0.23^{c}	-1.64b	-0.30^{b}	-1.95^{b}	0.221	0.608	<0.001
	6 months	1.24^{b}	0.32^{a}	0.26^{b}	-0,13^{a}	0.278	0.205	0.248
	7 months	2.01^{b}	-0.17^{a}	1.17^{b}	-0.68^{a}	0.264	0.089	<0.001
	8 months	4.55^{a}	1.28^{a}	4.48^{a}	0.32^{a}	0.416	0.314	<0.001
***p*-value**		<0.001	0.003	<0.001	0.001			
Yellowness (b*)	5 months	7.95	9.23	7.68ab	7.68	0.274	0.092	0.231
	6 months	7.99	11.01	6.24b	9.74	0.418	0.012	<0.001
	7 months	8.35	10.06	9.54^{a}	9.67	0.394	0.619	0.260
	8 months	10.18	11.29	8.96^{a}	10.33	0.426	0.206	0.151
***p*-value**		0.143	0.150	0.006	0.061			
Drip losses (%)	5 months	13.82^{a}	11.38^{a}	12.15^{a}	11.77^{a}	0.542	0.557	0.205
	6 months	7.86^{b}	7.52^{b}	7.05^{b}	7.01^{b}	0.218	0.146	0.666
	7 months	6.48^{b}	4.08^{c}	6.78^{b}	4.32^{c}	0.306	0.543	<0.001
	8 months	4.31^{c}	3.26^{c}	3.85^{c}	3.19^{c}	0.132	0.224	0.001
***p*-value**		<0.001	<0.001	<0.001	<0.001			

Abbreviations: SEM: standard error of the mean; values with different superscript letters (a, b, c) within the same column per fixed effect (age of slaughter) differ significantly (p< 0.05). The effect of interaction between "Breed" and "Gender" was not significant; therefore, significance is only presented for main effects.

Table S3. Chemical composition (g/100 g FM) and hydroxyproline content (mg/g dry weight) of breast meat at different slaughter ages (n = 8).

Items	Age at slaughter	*Bionda Piemontese*		*Bianca di Saluzzo*		SEM	*p*-value	
		Male	Female	Male	Female		Breed	Gender
Moisture	5 months	75.13^{a}	73.22^{ab}	74.98^{a}	73.38	0.227	0.984	<0.001
	6 months	74.63^{ab}	74.08^{a}	74.37^{ab}	73.82	0.119	0.254	0.020
	7 months	73.90^{bc}	72.53^{c}	73.55^{b}	73.06	0.162	0.740	0.002
	8 months	73.75^{c}	73.47^{ab}	73.79^{b}	73.42	0.119	0.998	0.192
***p*-value**		0.03	0.03	0.011	0.294			
Crude protein	5 months	26.13	24.30	26.66^{a}	25.20	0.325	0.235	0.010
	6 months	24.20	24.62	24.36^{c}	24.52	0.115	0.901	0.223
	7 months	24.73	25.18	25.96^{ab}	24.93	0.220	0.264	0.514
	8 months	24.95	24.82	25.31^{ab}	24.72	0.136	0.642	0.197
***p*-value**		0.071	0.489	0.006	0.330			
Crude fat	5 months	0.23	0.75^{b}	0.11^{b}	0.55^{b}	0.069	0.147	<0.001
	6 months	0.31	0.61^{b}	0.29^{a}	0.88^{ab}	0.087	0.429	0.009
	7 months	0.33	1.72^{a}	0.30^{a}	1.22^{a}	0.138	0.162	<0.001
	8 months	0.37	0.50^{b}	0.21^{ab}	0.35^{b}	0.038	0.033	0.056
***p*-value**		0.329	<0.001	0.046	0.014			
Ash	5 months	1.14	1.22	1.13^{b}	1.22	0.013	0.819	0.004
	6 months	1.17	1.20	1.15^{b}	1.15	0.008	0.048	0.301
	7 months	1.19	1.18	1.21^{a}	1.16	0.010	1.000	0.260
	8 months	1.21	1.20	1.18^{ab}	1.21	0.009	0.767	0.576
***p*-value**		0.250	0.769	0.036	0.065			
Hydroxy proline	5 months	1.49	2.33^{ab}	1.12^{c}	2.48	0.179	0.722	0.001
	6 months	1.95	1.84^{b}	1.98^{bc}	2.13	0.102	0.454	0.918
	7 months	2.75	3.02^{a}	3.06^{a}	3.04	0.223	0.500	0.956
	8 months	2.99	3.00^{a}	2.89^{ab}	3.16	0.169	0.903	0.668
***p*-value**		0.075	0.013	<0.001	0.107			

Abbreviations: FM: fresh matter; SEM: standard error of the mean; values with different superscript letters (a, b, c) within the same column per fixed effect (age of slaughter) differ significantly ($p < 0.05$). The effect of interaction between "Breed" and "Gender" was not significant; therefore, significance is only presented for main effects.

Table S4. Chemical composition (g/100 g FM) of thigh meat at different slaughter ages (n = 8).

**Items**	**Age at slaughter**	***Bionda Piemontese***		***Bianca di Saluzzo***		**SEM**	***p*-value**	
		**Male**	**Female**	**Male**	**Female**		**Breed**	**Gender**
Moisture	5 months	76.19^{a}	82.21^{a}	76.42^{a}	81.47^{a}	0.740	0.827	<0.001
	6 months	74.96^{b}	73.39^{b}	75.04^{b}	73.12^{b}	0.211	0.752	<0.001
	7 months	75.26^{b}	71.54^{b}	75.08^{b}	71.84^{b}	0.346	0.854	<0.001
	8 months	74.76^{b}	72.57^{b}	75.12^{b}	72.14^{b}	0.329	0.940	<0.001
***p*-value**		0.004	<0.001	0.003	<0.001			
Crude protein	5 months	26.85	26.88	27.13	27.89	0.303	0.308	0.526
	6 months	27.11	26.35	27.38	26.19	0.186	0.873	0.008
	7 months	27.58	28.15	27.81	29.33	0.298	0.416	0.232
	8 months	27.36	27.98	27.68	28.72	0.417	0.540	0.340
***p*-value**		0.682	0.359	0.747	0.018			
Crude fat extract	5 months	1.00^{b}	2.30^{c}	0.96^{c}	2.28c	0.136	0.859	<0.001
	6 months	2.15^{a}	4.33^{b}	2.55^{a}	4.91^{b}	0.274	0.194	<0.001
	7 months	1.65^{a}	6.92^{a}	1.79^{b}	6.14^{a}	0.460	0.325	<0.001
	8 months	2.03^{a}	4.28^{b}	1.84^{b}	4.01^{b}	0.220	0.246	<0.001
***p*-value**		<0.001	<0.001	<0.001	<0.001			
Ash	5 months	1.08	0.75^{b}	1.08	0.77^{c}	0.038	0.838	<0.001
	6 months	1.13	1.12^{a}	1.12	1.08^{ab}	0.009	0.227	0.121
	7 months	1.10	1.02^{a}	1.07	1.01^{b}	0.008	0.035	<0.001
	8 months	1.13	1.13^{a}	1.11	1.14^{a}	0.011	0.937	0.400
***p*-value**		0.060	<0.001	0.066	<0.001			

Abbreviations: FM: fresh matter; SEM: standard error of the mean; values with different superscript letters (a, b, c) within the same column per fixed effect (age of slaughter) differ significantly ($p < 0.05$). The effect of interaction between "Breed" and "Gender" was not significant; therefore, significance is only presented for main effects.

Table S5. Saturated fatty acid profile of breast meat at different slaughter ages (g/100g of total fatty acids) (n = 8).

Items	Age at slaughter	*Bionda Piemontese*		*Bianca di Saluzzo*		SEM	*p*-value	
		Male	Female	Male	Female		Breed	Gender
C14	5 months	0.36	0.60^{b}	0.13	0.41^{b}	0.049	0.014	0.003
	6 months	0.39	0.51^{b}	0.18	0.48^{b}	0.034	0.032	0.001
	7 months	0.41	0.86^{a}	0.32	0.66^{a}	0.049	0.042	<0.001
	8 months	0.51	0.59^{b}	1.02	0.50^{b}	0.191	0.549	0.571
***p*-value**		0.531	0.001	0.362	0.023			
C16	5 months	24.35^{b}	28.43^{bc}	24.72	26.36	0.580	0.427	0.427
	6 months	27.74^{a}	26.74^{c}	27.09	27.60	0.421	0.905	0.781
	7 months	30.05^{a}	33.27^{a}	27.07	29.10	0.656	0.003	0.023
	8 months	30.41^{a}	29.20^{b}	28.88	27.97	0.417	0.101	0.204
***p*-value**		0.001	<0.001	0.171	0.103			
C17	5 months	0.24	0.32^{ab}	0.16	0.26	0.024	0.133	0.072
	6 months	0.25	0.20^{c}	0.16	0.30	0.022	0.942	0.269
	7 months	0.48	0.41^{a}	0.21	0.40	0.039	0.062	0.375
	8 months	0.37	0.31^{b}	0.43	0.29	0.058	0.878	0.394
***p*-value**		0.123	0.003	0.352	0.106			
C18	5 months	12.79^{b}	12.77^{a}	14.06^{b}	12.73^{a}	0.249	0.207	0.168
	6 months	13.74^{b}	11.26^{b}	13.60^{b}	12.58^{a}	0.250	0.119	<0.001
	7 months	15.43^{a}	11.51^{b}	14.52^{b}	10.84^{b}	0.393	0.046	<0.001
	8 months	14.96^{a}	10.91^{b}	16.72^{a}	11.98^{a}	0.445	<0.001	<0.001
***p*-value**		<0.001	0.011	<0.001	0.006			
C24	5 months	1.56^{a}	1.06	2.04	1.58^{a}	0.097	0.004	0.005
	6 months	1.20^{ab}	1.27	1.52	0.90^{b}	0.127	0.924	0.283
	7 months	0.87^{bc}	0.66	1.12	0.35^{c}	0.103	0.894	0.015
	8 months	0.67^{c}	1.15	1.15	0.78^{b}	0.087	0.725	0.736
***p*-value**		0.002	0.232	0.065	<0.001			
ΣSFA	5 months	39.30^{c}	43.18^{b}	41.11^{b}	41.33	0.646	0.987	0.114
	6 months	43.33^{b}	39.98^{c}	42.55^{b}	41.87	0.487	0.547	0.035
	7 months	47.24^{a}	46.71^{a}	43.25^{b}	41.36	0.573	0.001	0.353
	8 months	46.93^{a}	42.16^{bc}	48.21^{a}	41.53	0.677	0.720	<0.001
***p*-value**		<0.001	<0.001	0.010	0.973			

Abbreviations: SFA: saturated fatty acids; SEM: standard error of the mean; values with different superscript letters (a, b, c) within the same column per fixed effect (age of slaughter) differ significantly ($p < 0.05$). The effect of interaction between "Breed" and "Gender" was not significant; therefore, significance is only presented for main effects.

Table S6. Monounsaturated fatty acid profile of breast meat at different slaughter ages (g/100g of total detected fatty acids) (n = 8).

Items	Age at slaughter	*Bionda Piemontese*		*Bianca di Saluzzo*		SEM	*p*-value	
		Male	Female	Male	Female		Breed	Gender
C16 :1n7	5 months	1.23	1.56^{b}	0.69	1.51^{b}	0.132	0.244	0.027
	6 months	1.07	1.95^{b}	1.23	1.80^{b}	0.134	0.986	0.006
	7 months	1.17	3.71^{a}	1.10	3.24^{a}	0.239	0.260	<0.001
	8 months	1.34	2.16^{b}	0.56	1.81^{b}	0.147	0.012	<0.001
***p*-value**		0.846	<0.001	0.056	<0.001			
C18 :1c9	5 months	25.46^{b}	31.20^{c}	23.34^{b}	29.87^{c}	0.804	0.157	<0.001
	6 months	28.40^{ab}	31.39^{c}	30.07^{a}	32.39^{bc}	0.706	0.340	0.063
	7 months	29.43^{a}	40.48^{a}	30.82^{a}	40.78^{a}	1.097	0.474	<0.001
	8 months	30.79^{a}	36.95^{b}	28.49^{a}	34.20^{b}	0.796	0.036	<0.001
***p*-value**		0.033	<0.001	0.001	<0.001			
C20: 1	5 months	0.20	0.18	0.06	0.28	0.030	0.756	0.071
	6 months	0.38	0.15	0.09	0.34	0.037	0.392	0.913
	7 months	0.23	0.26	0.26	0.29	0.034	0.681	0.681
	8 months	0.27	0.21	0.20	0.19	0.032	0.537	0.574
***p*-value**		0.224	0.605	0.229	0.403			
C24:1	5 months	0.58	0.64^{a}	0.98	0.53^{a}	0.072	0.289	0.159
	6 months	0.33	0.70^{a}	0.62	0.59^{a}	0.065	0.498	0.179
	7 months	0.56	0.15^{b}	0.42	0.21^{b}	0.088	0.808	0.085
	8 months	0.21	0.51^{a}	0.72	0.68^{a}	0.065	0.006	0.260
***p*-value**		0.384	0.013	0.090	0.006			
ΣMUFA	5 months	27.46	33.58^{c}	25.08^{b}	32.20^{c}	0.871	0.155	<0.001
	6 months	30.18	34.19^{c}	32.01^{a}	35.12^{bc}	0.812	0.381	0.029
	7 months	31.38	44.60^{a}	32.60^{a}	44.52^{a}	0.774	0.674	<0.001
	8 months	32.62	39.83^{b}	29.97^{a}	36.89^{b}	0.902	0.033	<0.001
***p*-value**		0.087	<0.001	0.003	<0.001			

Abbreviations: MUFA: monounsaturated fatty acids; SEM: standard error of the mean; values with different superscript letters (a, b, c) within the same column per fixed effect (age of slaughter) differ significantly ($p< 0.05$). The effect of interaction between "Breed" and "Gender" was not significant; therefore, significance is only presented for main effects.

Table S7. Polyunsaturated fatty acid profile of breast meat at different slaughter ages (g/100g of total detected fatty acids) (n = 8).

Items	Age at slaughter	*Bionda Piemontese*		*Bianca di Saluzzo*		SEM	*p*-value	
		Male	Female	Male	Female		Breed	Gender
C18 :2n6	5 months	15.18	13.07[a]	15.00	13.47	0.476	0.908	0.063
	6 months	12.97	13.95[a]	11.47	11.75	0.369	0.011	0.359
	7 months	11.71	4.71[b]	12.69	9.49	0.762	0.015	<0.001
	8 months	13.67	11.90[a]	13.37	11.22	0.567	0.666	0.094
***p*-value**		0.256	<0.001	0.056	0.060			
C20 :2n6	5 months	0.32	0.16[ab]	0.19	0.24	0.035	0.700	0.438
	6 months	0.25	0.11[b]	0.37	0.13	0.369	0.211	0.002
	7 months	0.22	0.03[b]	0.21	0.12	0.030	0.458	0.012
	8 months	0.23	0.28[a]	0.39	0.18	0.037	0.735	0.276
***p*-value**		0.631	0.009	0.224	0.376			
C20: 4n6	5 months	10.19[a]	6.86[a]	12.67[a]	8.06[a]	0.578	0.047	<0.001
	6 months	7.72[a]	7.35[a]	8.32[b]	6.24[ab]	0.501	0.803	0.243
	7 months	4.69[b]	0.65[b]	5.74[b]	1.84[c]	0.610	0.282	0.001
	8 months	2.81[b]	1.47[b]	1.48[c]	4.63[b]	0.483	0.313	0.320
***p*-value**		<0.001	<0.001	<0.001	<0.001			
Σn6 PUFA	5 months	25.69[a]	20.09[a]	27.85[a]	21.77[a]	0.909	0.217	0.004
	6 months	20.94[b]	21.43[a]	20.15[b]	18.25[ab]	0.714	0.177	0.625
	7 months	16.62[b]	5.41[c]	18.64[b]	11.48[c]	1.240	0.028	<0.001
	8 months	16.72[b]	13.65[b]	15.24[b]	16.03[bc]	0.822	0.792	0.503
***p*-value**		0.001	<0.001	<0.001	0.001			
C18 :3n3	5 months	0.06	0.11[b]	0.00[b]	0.15	0.018	0.759	0.004
	6 months	0.06	0.11[b]	0.03[b]	0.13	0.021	0.840	0.109
	7 months	0.09	0.10[b]	0.14[a]	0.19	0.020	0.109	0.506
	8 months	0.12	0.54[a]	0.02[b]	0.20	0.056	0.024	0.003
***p*-value**		0.597	0.001	0.002	0.742			
C20 :5n3	5 months	1.21[a]	0.36	0.71	0.40	0.201	0.571	0.159
	6 months	0.22[b]	0.44	0.61	0.62	0.158	0.389	0.735
	7 months	0.06[b]	0.01	0.50	0.01	0.061	0.039	0.014
	8 months	0.31[b]	0.12	1.00	0.30	0.166	0.193	0.179
***p*-value**		0.047	0.542	0.853	0.406			
C22 :5n3	5 months	1.08[a]	0.65[a]	1.25	0.70[a]	0.092	0.513	0.007
	6 months	0.60[b]	0.52[a]	0.85	0.54[a]	0.061	0.269	0.114
	7 months	0.40[b]	0.05[b]	0.77	0.15[b]	0.087	0.124	0.003
	8 months	0.36[b]	0.27[b]	0.84	0.49[a]	0.166	0.004	0.060
***p*-value**		0.004	<0.001	0.353	0.005			
C22 :6n3	5 months	0.95[a]	0.87[ab]	1.14	0.66[ab]	0.086	0.956	0.104
	6 months	0.56[b]	1.15[a]	0.72	0.73[ab]	0.076	0.347	0.036
	7 months	0.38[b]	0.08[c]	0.61	0.18[b]	0.071	0.199	0.007

	8 months	0.31[b]	0.49[bc]	0.80	1.13[a]	0.127	0.027	0.296
***p*-value**		0.008	<0.001	0.279	0.041			
Σn3 PUFA	5 months	3.31[a]	1.98 [a]	3.11	1.92[a]	0.280	0.805	0.028
	6 months	1.45[b]	2.22[a]	2.21	2.03[a]	0.209	0.511	0.493
	7 months	0.93[b]	0.24[b]	2.03	0.54[b]	0.168	0.008	<0.001
	8 months	1.10[b]	1.42[a]	2.66	2.11[a]	0.283	0.050	0.837
***p*-value**		0.002	<0.001	0.641	0.021			
ΣPUFA	5 months	29.01[a]	22.08[a]	30.96[a]	23.69[a]	1.041	0.303	0.002
	6 months	22.39[b]	23.63[a]	22.36[b]	20.15[ab]	0.829	0.305	0.774
	7 months	17.56[b]	5.63[c]	20.67[b]	11.98[c]	1.370	0.019	<0.001
	8 months	17.82[b]	15.08[b]	17.90[b]	18.14[b]	0.877	0.386	0.487
***p*-value**		<0.001	<0.001	0.001	<0.001			

Abbreviations: PUFA: polyunsaturated fatty acids; SEM: standard error of the mean; values with different superscript letters (a, b, c) within the same column per fixed effect (age of slaughter) differ significantly ($p < 0.05$). The effect of interaction between "Breed" and "Gender" was not significant; therefore, significance is only presented for main effects.

Table S8. Saturated fatty acid profile of thigh meat at different slaughter ages (g/100g of total fatty acids) (n = 8).

Items	Age at slaughter	*Bionda Piemontese*		*Bianca di Saluzzo*		SEM	*p*-value	
		Male	Female	Male	Female		Breed	Gender
C14	5 months	0.97[a]	1.26	0.86[a]	1.01	0.049	0.038	0.015
	6 months	0.97[a]	1.09	0.88[a]	0.99	0.022	0.013	0.005
	7 months	0.84[ab]	1.08	0.71[b]	1.05	0.033	0.071	<0.001
	8 months	0.80[b]	1.08	0.62[b]	0.96	0.035	<0.001	<0.001
***p*-value**		0.043	0.250	<0.001	0.470			
C16	5 months	32.53	36.68	30.53[ab]	32.90[b]	0.524	<0.001	<0.001
	6 months	33.27	35.58	32.16[a]	34.84[ab]	0.411	0.199	0.001
	7 months	32.96	36.27	31.42[a]	36.42[a]	0.486	0.277	<0.001
	8 months	31.62	36.42	29.05[b]	34.39[ab]	0.582	0.001	<0.001
***p*-value**		0.494	0.542	0.002	0.023			
C17	5 months	0.45	0.38	0.51	0.47	0.018	0.024	0.090
	6 months	0.43	0.39	0.44	0.45	0.025	0.509	0.813
	7 months	0.47	0.45	0.44	0.39	0.011	0.029	0.057
	8 months	0.43	0.49	0.43	0.32	0.021	0.031	0.469
***p*-value**		0.822	0.139	0.263	0.096			
C18	5 months	18.83[c]	13.12	21.33	14.64[a]	0.696	0.017	<0.001
	6 months	19.20[bc]	12.44	18.09	13.45[ab]	0.625	0.946	<0.001
	7 months	21.35[a]	12.36	19.45	12.00[b]	0.839	0.168	<0.001
	8 months	20.92[ab]	12.50	21.05	12.86[ab]	0.807	0.709	<0.001
***p*-value**		0.029	0.685	0.132	0.045			
C24	5 months	0.67	0.57[c]	0.50	0.50[b]	0.045	0.205	0.608
	6 months	0.31	0.96[bc]	0.76	0.88[ab]	0.076	0.156	0.006
	7 months	0.53	1.25[ab]	0.66	1.01[a]	0.090	0.734	0.002
	8 months	0.72	1.50[a]	0.62	1.14[a]	0.089	0.088	<0.001
***p*-value**		0.052	0.001	0.378	0.022			
ΣSFA	5 months	53.45[b]	52.01	53.73	49.52	0.537	0.248	0.005
	6 months	54.18[ab]	50.47	52.34	50.61	0.536	0.388	0.009
	7 months	56.15[a]	51.40	52.69	50.87	0.573	0.039	0.001
	8 months	54.49[ab]	51.99	51.77	49.67	0.562	0.017	0.028
***p*-value**		0.148	0.324	0.657	0.798			

Abbreviations: SFA: saturated fatty acids; SEM: standard error of the mean; values with different superscript letters (a, b, c) within the same column per fixed effect (age of slaughter) differ significantly ($p < 0.05$). The effect of interaction between "Breed" and "Gender" was not significant; therefore, significance is only presented for main effects.

Table S9. Monounsaturated fatty acid profile of thigh meat at different slaughter ages (g/100g of total fatty acids) (n = 8).

Items	Age at slaughter	*Bionda Piemontese*		*Bianca di Saluzzo*		SEM	*p*-value	
		Male	Female	Male	Female		Breed	Gender
C16 :1n7	5 months	3.07^{a}	4.67	2.53^{ab}	3.88	0.196	0.022	<0.001
	6 months	2.95^{a}	4.46	3.27^{a}	4.24	0.174	0.865	<0.001
	7 months	2.20^{b}	4.24	2.53^{ab}	4.63	0.223	0.159	<0.001
	8 months	2.47^{ab}	4.61	1.97^{b}	3.98	0.225	0.026	<0.001
***p*-value**		0.019	0.762	0.026	0.142			
C18 :1c9	5 months	34.84	36.63	32.62^{b}	37.73	0.509	0.482	<0.001
	6 months	36.44	38.98	37.41^{a}	40.04	0.511	0.288	0.011
	7 months	33.68	39.25	36.96^{a}	40.25	0.602	0.016	<0.001
	8 months	34.97	37.88	34.57^{ab}	40.18	0.575	0.269	<0.001
***p*-value**		0.102	0.087	0.008	0.137			
C20: 1	5 months	0.40	0.39	0.53	0.55^{a}	0.024	0.002	0.849
	6 months	0.46	0.38	0.55	0.41^{b}	0.023	0.153	0.011
	7 months	0.37	0.36	0.42	0.34^{bc}	0.034	0.840	0.569
	8 months	0.38	0.26	0.48	0.26^{c}	0.036	0.466	0.021
***p*-value**		0.686	0.344	0.548	<0.001			
C24:1	5 months	0.02^{b}	0.14	0.30	0.14	0.034	0.032	0.705
	6 months	0.37^{a}	0.25	0.18	0.19	0.041	0.138	0.468
	7 months	0.11^{b}	0.23	0.16	0.13	0.033	0.696	0.568
	8 months	0.22^{ab}	0.31	0.13	0.14	0.036	0.065	0.479
***p*-value**		0.014	0.442	0.371	0.899			
ΣMUFA	5 months	38.34^{ab}	41.83	35.98^{c}	42.31	0.643	0.321	<0.001
	6 months	40.22^{a}	44.07	41.42^{a}	44.88	0.647	0.394	0.004
	7 months	36.36^{b}	44.08	40.07^{ab}	45.35	0.774	0.013	<0.001
	8 months	38.04^{ab}	43.07	37.15^{bc}	44.56	0.759	0.777	<0.001
***p*-value**		0.049	0.230	0.020	0.148			

Abbreviations: MUFA: monounsaturated fatty acids; SEM: standard error of the mean; values with different superscript letters (a, b, c) within the same column per fixed effect (age of slaughter) differ significantly ($p < 0.05$). The effect of interaction between "Breed" and "Gender" was not significant; therefore, significance is only presented for main effects.

Table S10. Polyunsaturated fatty acid profile of thigh meat at different slaughter ages (g/100g of total fatty acids) (n = 8).

Items	Age at slaughter	*Bionda Piemontese*		*Bianca di Saluzzo*		SEM	*p*-value	
		Male	Female	Male	Female		Breed	Gender
C18 :2n6	5 months	4.59	2.98[a]	4.92	3.07[a]	0.316	0.716	0.006
	6 months	4.10	2.13[b]	3.27	2.16[b]	0.225	0.275	<0.001
	7 months	4.12	1.72[b]	4.21	1.35[c]	0.280	0.645	<0.001
	8 months	4.69	2.03[b]	6.48	2.11[b]	0.495	0.232	<0.001
***p*-value**		0.719	0.001	0.110	<0.001			
C20 :2n6	5 months	0.03	0.02[a]	0.02	0.02	0.007	0.781	0.968
	6 months	0.04	0.00[b]	0.01	0.00	0.006	0.236	0.065
	7 months	0.00	0.00[b]	0.01	0.00	0.002	0.165	0.165
	8 months	0.03	0.00[b]	0.04	0.01	0.010	0.755	0.112
***p*-value**		0.595	0.004	0.438	0.290			
C20: 4n6	5 months	0.46	0.49[a]	1.80[a]	1.13	0.210	0.017	0.427
	6 months	0.43	0.13[b]	0.44[b]	0.18	0.046	0.719	0.002
	7 months	0.49	0.12[b]	0.45[b]	0.07	0.058	0.655	0.001
	8 months	0.66	0.11[b]	0.37[b]	0.45	0.089	0.882	0.168
***p*-value**		0.702	0.041	0.003	0.063			
Σn6 PUFA	5 months	5.08	3.49[a]	6.73[a]	4.23[a]	0.358	0.056	0.002
	6 months	4.57	2.26[b]	3.72[b]	2.35[b]	0.271	0.389	<0.001
	7 months	4.61	1.84[b]	4.67[ab]	1.42[c]	0.323	0.624	<0.001
	8 months	5.39	2.14[b]	6.90[a]	2.56[b]	0.502	0.207	<0.001
***p*-value**		0.674	0.001	0.045	<0.001			
C18 :3n3	5 months	0.08	0.09	0.22	0.15	0.025	0.057	0.514
	6 months	0.19	0.08	0.13	0.10	0.025	0.782	0.188
	7 months	0.17	0.12	0.12	0.06	0.024	0.293	0.240
	8 months	0.13	0.09	0.05	0.03	0.018	0.061	0.468
***p*-value**		0.652	0.800	0.170	0.104			
C20 :5n3	5 months	0.00	0.09	0.00	0.07	0.022	0.793	0.079
	6 months	0.00	0.29	0.07	0.06	0.064	0.542	0.281
	7 months	0.00	0.13	0.00	0.05	0.026	0.484	0.085
	8 months	0.11	0.27	0.00	0.01	0.056	0.102	0.461
***p*-value**		0.407	0.757	0.407	0.693			
C22 :5n3	5 months	0.00	0.00	0.04[b]	0.15	0.028	0.103	0.335
	6 months	0.00	0.11	0.00[b]	0.00	0.029	0.326	0.326
	7 months	0.00	0.00	0.00[b]	0.00	-	-	-
	8 months	0.00	0.00	0.62[a]	0.00	0.097	0.094	0.094
***p*-value**		-	0.407	0.055	0.132			
C22 :6n3	5 months	0.24[a]	0.00	0.00[b]	0.12	0.041	0.441	0.441
	6 months	0.00[b]	0.12	0.00[b]	0.00	0.030	0.326	0.326
	7 months	0.00[b]	0.00	0.00[b]	0.00	-	-	-

	8 months	0.00[b]	0.00	0.94[a]	0.00	0.146	0.090	0.090
***p*-value**		0.028	0.407	0.043	0.132			
Σn3 PUFA	5 months	0.32	0.19	0.25	0.51[a]	0.059	0.263	0.631
	6 months	0.19	0.60	0.20	0.16[b]	0.081	0.187	0.238
	7 months	0.17	0.25	0.12	0.12[b]	0.037	0.257	0.667
	8 months	0.24	0.36	1.60	0.04[b]	0.242	0.262	0.126
***p*-value**		0.753	0.369	0.078	0.011			
ΣPUFA	5 months	5.40	3.67[a]	6.99[ab]	4.74[a]	0.379	0.053	0.005
	6 months	4.76	2.87[b]	3.93[c]	2.51[bc]	0.267	0.201	0.001
	7 months	4.79	2.09[b]	4.80[bc]	1.54[c]	0.319	0.458	<0.001
	8 months	5.63	2.50[b]	8.50[a]	2.60[b]	0.564	0.052	<0.001
***p*-value**		0.623	0.002	0.004	<0.001			

Abbreviations: PUFA: polyunsaturated fatty acids; SEM: standard error of the mean; values with different superscript letters (a, b, c) within the same column per fixed effect (age of slaughter) differ significantly ($p < 0.05$). The effect of interaction between "Breed" and "Gender" was not significant; therefore, significance is only presented for main effects.
